# Editorial: Chemical testing using new approach methodologies (NAMs)

**DOI:** 10.3389/ftox.2022.1048900

**Published:** 2022-11-10

**Authors:** Andreas O. Stucki, Amy J. Clippinger, Tala R. Henry, Carole Hirn, Todd J. Stedeford, Claire Terry

**Affiliations:** ^1^ PETA Science Consortium International e.V, Stuttgart, Germany; ^2^ Office of Pollution Prevention and Toxics, US Environmental Protection Agency, Washington, DC, United States; ^3^ Scientific and Regulatory Affairs, JT International SA, Geneva, Switzerland; ^4^ Bergeson & Campbell PC, Washington, DC, United States; ^5^ Corteva Agriscience, Versoix, Switzerland

**Keywords:** new approach methodologies (NAMs), chemical toxicity testing, *in vitro*, *in silico*, non-animal methods

In 2007, the U.S. National Research Council (NRC) introduced a novel approach to assure the safety of chemicals based on human-relevant mechanisms of toxicity in their landmark publication “Toxicity Testing in the 21^st^ Century: A Vision and a Strategy” (NRC, 2007). As written by Andersen and Krewski (2009), member and chair of the NRC Committee on Toxicity Testing and Assessment of Environmental Agents, the vision detailed “a not-so-distant future in which virtually all routine testing would be conducted in human cells or lines *in vitro*”. This Research Topic in Frontiers in Toxicology’s section on “*In Vitro* Toxicology” is devoted to highlighting research and methods that continue to advance this vision and to informing readers of the regulatory frameworks where these technologies may be used to inform regulatory filings and decisions.

Although no unanimous definition of new approach methodologies (NAMs) exists, NAMs are often defined as any technology, methodology, approach, or combination thereof that can provide information on chemical hazard and risk assessment without the use of animals, including *in silico*, *in chemico*, *in vitro*, and *ex vivo* approaches (ECHA, 2016; EPA, 2018; Stucki et al.). NAMs are not necessarily newly developed methods, rather, it is their application to regulatory decision making or replacement of a conventional testing requirement that is new. In this Research Topic, we are pleased to present on contributions to NAMs development and go a step further by bridging the science behind NAMs with the regulatory frameworks under which NAMs may be used, and in some cases, the regulatory impediments for their use.

Three original research articles on respiratory toxicity provide NAMs for evaluating specific regions of the respiratory tract, such as an adverse outcome pathway (AOP) for impairment of mucociliary clearance in the tracheobronchial region (Luettich et al.) that can be used to design in vitro testing strategies. Sengupta et al. present a lung-on-a-chip with a newly established cell line from epithelial cells from the alveolar regions. Zhang et al. introduce approaches for performing in vitro to in vivo extrapolations (IVIVE) for inhaled mixtures. These NAMs may be combined to provide a more complete understanding of the potential for inhaled chemical substances to cause adverse effects along the respiratory tract, and importantly, for deriving in vivo equivalent concentrations for quantitative risk assessments.

The original research articles also include investigations on the utility of cardiomyocytes derived from induced pluripotent stem cells as a screening tool for potential cardiotoxicity of new tobacco and other nicotine-containing products (Simms et al.), as well as investigations on potentially expanding the applicability domain of the Organisation for Economic Co-operation and Development’s (OECD)’s Guideline 497 on Defined Approaches for Skin Sensitisation from individual substances to pesticide formulations (Strickland et al.).

Beyond the original research articles, this Research Topic includes a methods article that advances our understanding of mitochondrial dysfunction and oxidative stress, and its role in acute and “low noise” chronic toxic effects (Loric and Conti).

Few would argue that the research and methods development on NAMs that has taken place since the NRC published its 2007 vision is nothing short of impressive. However, regulatory acceptance is a fundamental requirement for advancing the use and application of NAMs. This Research Topic provides three contributions that aid with understanding various regulatory framework where NAMs may be used and how NAMs were applied already (Miller-Holt et al. and Stucki et al.), as well as perspectives from Canadian regulators on their approaches for utilizing NAMs (Bhuller et al.).

Collectively, these articles showcase available test methods, how to design testing strategies using defined approaches or AOPs, or the current opportunities to use NAMs and proposals to help further advance their use and regulatory acceptance. The contributions in this Research Topic highlight the promise of answering “Yes” to the question “Is Animal Testing Overrated?“, a question posed by the U.S. Environmental Protection Agency nearly 4 decades ago (EPA, 1984). Realizing the full potential of NAMs will only be achieved, however, through continued publications, discussion, and transparency about new testing approaches as well as continued cross-sector partnerships and educational opportunities for stakeholders to gain confidence in NAMs (van der Zalm, 2022). Ultimately, these activities are leading to improved toxicology testing paradigms that better align with the NRC’s 2007 vision of harnessing modern technology and our current understanding of human biology to protect human health, while avoiding testing on animals.
